# Editorial: Robotics in extreme environments, volume II

**DOI:** 10.3389/frobt.2023.1337681

**Published:** 2023-11-24

**Authors:** Chie Takahashi, Barry Lennox, Claudio Semini, Manuel Giuliani, Young Soo Park, William R. Hamel

**Affiliations:** ^1^ Department of Psychology, University of Cambridge, Cambridge, United Kingdom; ^2^ Manchester Centre for Robotics and AI, The University of Manchester, Manchester, United Kingdom; ^3^ Dynamic Legged Systems Lab, Istituto Italiano di Tecnologia (IIT), Genoa, Italy; ^4^ Faculty of Electrical Engineering, Kempten University of Applied Sciences, Kempten, Germany; ^5^ Robotics and Remote Systems, Argonne National Laboratory, Illinois, United States; ^6^ Mechanical, Aerospace, and Biomedical Engineering Department, University of Tennessee, Knoxville, United States

**Keywords:** robotics, sensing, manipulation, autonomous systems, remote operation, human-robot interaction

## Introduction

**FIGURE 1:**
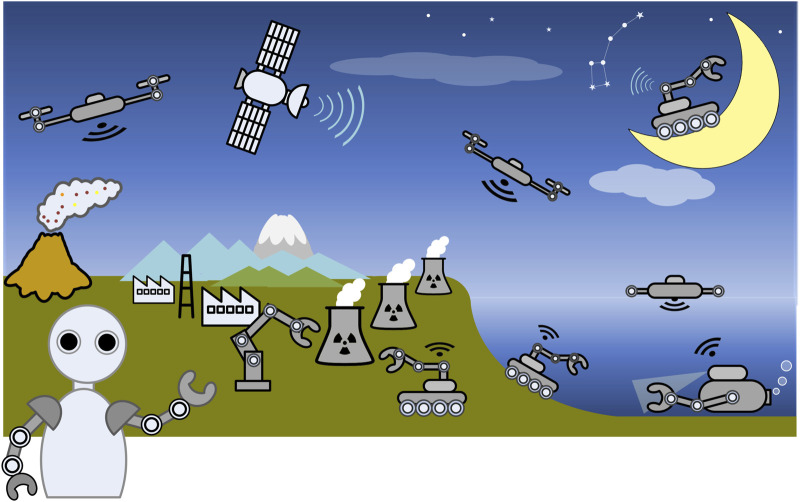
A Conceptual Overview of Research Fields related to Robotics in Extreme Environments. This figure illustrates the breadth of the Research Topic, encompassing diverse fields such as underwater applications, deployments in nuclear facilities, and space applications. (Note: some icons were originally sourced from Google’s ‘Free to Use’ section.).

This is the second volume of work, published by Frontiers in Robotics and AI, related to advances in the development and deployment of robotics technology in extreme environments, such as those experienced in offshore, nuclear and facilities containing high-magnetic fields. The articles published in this Research Topic demonstrate how robots can now be used to reliably provide inspection and mapping capabilities in these hazardous environments. Figure 1 shows a conceptual overview of the research areas related to this field.

Robotic solutions for hazardous environments significantly differ from those in factory automation, as the former must navigate unstructured and unpredictable surroundings while facing harsh conditions. Historically, robotic development was complex and often unaffordable for smaller R&D groups and industries. Recent advancements in components, digital technologies, and substantial investments in robotics and AI research over the last few years have enabled the development of robotic systems capable of performing tasks that were previously considered either impossible or required manual intervention, thus reducing the need to expose individuals to high-risk situations. This has required many advances in robotics and AI technologies to be made, delivering a range of cross-domain engineering solutions.

We initially raised this Research Topic to bring together the latest cutting-edge research in the field, to deepen the current understanding and to share some of the research challenges (refer to the first volume). In this second volume, we highlight some of the many advances that continue to be made in this field, as well as introduce new application domains. The Research Topic features four articles that provide case studies where robots are used for radiation mapping and surveillance, inspection of equipment within the European Organization for Nuclear Research (CERN) facility, and the identification of high-altitude wind conditions for improved weather forecasting and predicting pollution events in volcanic eruptions.

## Overview of the contents of the e-book structured

### CARMA II: A ground vehicle for autonomous surveying of alpha, beta and gamma radiation

Abadi et al. present some of the most recent results of a research collaboration between University of Manchester and Sellafield Ltd. (United Kingdom) that focusses on the introduction of robotics towards autonomous radiation monitoring of nuclear facilities. In this work, the CARMA II robot, the second version of the continuous autonomous radiation-monitoring assistant, is presented and experimentally demonstrated in a representative environment. Unlike other radiation monitoring robots, CARMA II can simultaneously map and avoid alpha, beta and gamma radiation. The navigation system of the robot receives a costmap that combines obstacles, as well as alpha, beta and gamma radiation that allows the robot to replan its path to avoid spreading contamination with its wheels.

### Airborne gamma-ray mapping using fixed-wing vertical take-off and landing (VTOL) uncrewed aerial vehicles

Woodbridge et al. conducted a drone survey over a uranium mine using a modified fixed-wing vertical take-off and landing UAV (uncrewed aerial vehicle), which was designed to detect radiation in the environment. The study demonstrated the system effectiveness by providing rapid airborne radiation mapping over large areas and comparing with several conventional systems and other UAV-based surveys. Although it was a prototype, their results indicate that the system and the technology would be beneficial for environmental mapping and incident management applications, moreover in emergency scenarios and unsafe situations such as natural and man-made disasters.

### Mission analysis, dynamics and robust control of an indoor blimp in a CERN detector magnetic environment

Mazzei et al. describe some of the research that has been completed within an on-going research programme at CERN to support the inspection and maintenance of some of its facilities. Access to these facilities can be restricted because of the high magnetic and radiation fields that are present. In this work, a robotic, lighter than air aerial vehicle (a blimp), is developed that can inspect facilities, with minimal risk of damaging them. The paper presents and compares several control techniques that can be used to enable the blimp to follow a reference trajectory. The blimp is observed to successfully follow a pre-specified trajectory, demonstrating the feasibility of using this technology to provide accurate inspection of facilities such as this in the future.

### High-altitude vertical wind profile estimation using multirotor vehicles

Autonomous aerial systems are increasingly being used in wide ranges of physical sizes and application domains. McConville and Richardson present an innovative use of multirotor vehicle dynamics to measure wind speed and direction at high altitudes such as those associated with the extreme updrafts of volcanic activity. Local wind changes disturb the vehicle dynamic behavior while flying in the updrafts. An algorithm which uses a vehicle dynamics model and real-time flight sensor data, particularly climbing rates, is then used to estimate wind speed and direction. Results from field experiments in Guatemala, which verify the performance of the new approach, are included.

## Conclusion

This volume of the Frontier journal presents comprehensive case examples of robotic evolution, including the development of software frameworks and the rapid prototyping of various robotic systems in remote and hazardous environments. The four presented papers demonstrate the field’s maturity since the first volume. While these papers focus on practical applications for inspection and monitoring using mobile and aerial robots, it has to be mentioned that there have also been considerable advances in teleoperated, human-in-the-loop robots for mobile and stationary manipulation. The increased application of robotics specifically in the nuclear industry evidences the growing trust of industry partners in the maturity of the technology. In the presented state, robotics applications in extreme environments often still have a human operator closely observing the robot. Moving forward, we anticipate that we will see more and more fully autonomous robots being used in extreme environments, initially for monitoring operations, later also for maintenance and repair, especially for critical infrastructure.

